# Evaluating the Linguistic Appropriateness and Cultural Sensitivity of a Self-Report System for Spanish-Speaking Patients with Cancer

**DOI:** 10.1155/2014/702683

**Published:** 2014-06-19

**Authors:** Cindy Tofthagen, Barbara Halpenny, Maribel Melendez, Laura Gonzalez, Veronica Sanchez Varela, Rosalyn Negrón, Donna L. Berry

**Affiliations:** ^1^University of South Florida, 4202 E Fowler Avenue, Tampa, FL 33620, USA; ^2^University of Massachusetts Boston, 100 Morrissey Boulevard, Boston, MA 02125, USA; ^3^Dana-Farber Cancer Institute, 450 Brookline Avenue, Boston, MA 02215, USA; ^4^University of Central Florida, 4000 Central Florida Boulevard, Orlando, FL 32816, USA; ^5^California Department of State Hospitals, P.O. Box 2297, Vacaville, CA 95696, USA

## Abstract

Spanish speakers in the United States encounter numerous communication barriers during cancer treatment. Communication-focused interventions may help Spanish speakers communicate better with healthcare providers and manage symptoms and quality of life issues (SQOL). For this study, we developed a Spanish version of the electronic self-report assessment for cancer (ESRA-C), a web-based program that helps people with cancer report, track, and manage cancer-related SQOL. Four methods were used to evaluate the Spanish version. Focus groups and cognitive interviews were conducted with 51 Spanish-speaking individuals to elicit feedback. Readability was assessed using the Fry readability formula. The cultural sensitivity assessment tool was applied by three bilingual, bicultural reviewers. Revisions were made to personalize the introduction using a patient story and photos and to simplify language. Focus group participants endorsed changes to the program in a second round of focus groups. Cultural sensitivity of the program was scored unacceptable ($\bar{x}=3.0$) for audiovisual material and acceptable ($\bar{x}=3.0$) for written material. Fry reading levels ranged from 4th to 10th grade. Findings from this study provide several next steps to refine ESRA-C for Spanish speakers with cancer.

## 1. Introduction

Hispanics are the fastest growing minority group in the United States (US), currently comprising over 15% of the total population [1]. Approximately, 38% of Hispanics in the United States are Spanish-language dominant and another 38% are bilingual [2]. Hispanics are a diverse group with respect to socioeconomic characteristics, level of acculturation, national origin, and heritage. These factors have all been linked to cancer [3, 4] and other health outcomes [5–9]. Cancer is the leading cause of death among Hispanics in the US [10]. Spanish-language dominant individuals face barriers to communicating with healthcare providers and have less access to Spanish information, both in print and on the Internet, than do English-speaking patients [11].

The number of Hispanics who utilize the Internet is increasing; however, only 1/3 of Spanish-language dominant Hispanics use the Internet [12]. Hispanics born outside the US, who are older, of lower educational levels, less proficient in English, or of Mexican nationality, are less likely to use the Internet [12]. Hispanics are also more likely to report dissatisfaction with medical care, perhaps because of challenges communicating with healthcare providers [13–15].

Studies of cancer treatment-related symptoms in Hispanic patients regularly report higher symptom burden, especially depression and pain [16–18]. This may be attributed to culture, family beliefs, and religion (e.g., a stoic attitude toward pain) [19, 20] or physician underestimation of the problem [17, 21, 22]. Poor provider-patient communication adversely impacts the ability of patients to communicate their experiences, understand, and adhere to treatment recommendations [23]. A survey of 624 key opinion leaders around the country, conducted by the National Cancer Institute's National Hispanic/Latino Cancer Network, identified patient-doctor communication as one of five cancer prevention and control issues of greatest significance to the Hispanic population [24].

Symptoms and quality of life issues (SQOL) are important aspects of cancer and treatment. These SQOL issues can be challenging for patients and healthcare providers to communicate about, even when speaking the same language [25]. Assessment and management of symptoms are fundamental to quality care in oncology, yet emphasis on medical treatments and procedures, time constraints, and perceived lack of effective treatments for SQOL may limit time spent in discussion [26]. The electronic self-report assessment for cancer (ESRA-C) is a web-based program developed to help patients monitor and manage SQOL and communicate more effectively with their healthcare providers [26–28]. The program records severity of patient-reported SQOL and provides a summary to clinicians about which SQOL are causing distress. The ESRA-C allows patient users to self-monitor SQOL and receive self-care education and coaching on how to communicate troublesome issues. Two multisite, randomized clinical trials demonstrated that use of ESRA-C increased discussion of distressing SQOL between English-speaking patients with various types of cancer and providers [26] and effectively reduced cancer-related symptom distress, particularly among individuals greater than 50 years old [29].

Family caregivers play an important role in managing cancer-related side effects. Hispanic cultures have long been recognized for collectivistic and multigenerational households where elders are cared for and respected [41].* Personalismo* has been described as a core value in Hispanic cultures and places a high value on informal and supportive relationships with others [42]. Similarly,* familismo* is a core value that emphasizes importance of extended family and a responsibility to care for and support each other. The reliance on family caregivers and the complex challenges they and their caregivers face are important to honor and understand [43]. Because Spanish-speaking patients with cancer cared for in an English-speaking institution are at risk for poor patient-provider communication, we planned a Spanish version of ESRA-C that could be especially useful for Spanish speakers with cancer, helping to bridge the communication gap with healthcare providers, providing information on expected side effects of cancer treatment, and allowing them to monitor and care for changes in SQOL over time. The purpose of this study was to develop a linguistically appropriate and culturally sensitive Spanish version of the electronic self-report assessment for cancer (ESRA-C) with input from Spanish-speaking participants from a variety of backgrounds. We sought to accomplish this by (1) holding focus groups with patients and family caregivers to elicit information about the appeal and cultural sensitivity of ESRA-C Spanish; (2) conducting cognitive interviews with Spanish-speaking participants to identify any issues with readability or cultural sensitivity in the translated instruments; (3) assessing the cultural sensitivity of ESRA-C Spanish using the cultural sensitivity assessment tool (CSAT) [40], a tool that has been used by expert reviewers to evaluate cancer websites; and (4) employing the Fry formula [44, 45] to target the 6th grade reading level recommended for patient education materials in cancer care settings [46].

## 2. Materials and Methods

### 2.1. Samples and Settings

Participants in this study were recruited from two sites: Dana-Farber Cancer Institute in Boston, Massachusetts, and a community medical oncology practice with a primarily Hispanic patient population in Tampa, Florida. To be eligible for participation, individuals had to be at the age of 18 or older; Spanish-language-dominant; self-identified Hispanic ethnicity; receiving cancer treatment, a cancer survivor, or a caregiver of someone with cancer. Caregivers were defined for this study as a family member or friend who assists with medical care in any capacity and were included for several reasons: increasing the sample size; importance of family in Hispanic culture; and knowledge of the treatment and experiences of their family member.

Internal Review Board protocol approvals from both the Dana-Farber/Harvard Cancer Center and the University of South Florida were obtained prior to recruitment. In Boston, potential participants were recruited through electronic medical records and also through a Dana-Farber Latino Patient and Caregiver conference. Interested conference attendees were asked to provide contact information. Potentially eligible patients were mailed an invitation letter with an opt-out response form. A research assistant telephoned patients who did not opt out. The study staff obtained approval for participation from patients' oncology providers. Patients were given the opportunity to ask a caregiver to participate. In Tampa, eligible participants were identified and referred to the study by the oncologist and then contacted by study staff to determine interest in participation and obtain consent.

### 2.2. Key Informant Panel Meetings

The study team met three times with a key informant panel from the Dana-Farber interpreter, patient navigator, social work, and outreach services. These four bilingual, Hispanic panel members reviewed the existing English ESRA-C website and provided suggestions for focus group discussion and enhancements to ESRA-C. The study team implemented the panel's recommendations prior to the first round of focus groups by (1) adding information about members of the healthcare team and respective roles; (2) using photographs to illustrate interaction with the care team; and (3) adding pop-up definitions to assist lower literacy patients in understanding terminology on the website. The panel also recommended study of participant understanding of questionnaire items and responses that are included in ESRA-C. Suggestions by the panel that were outside the scope of our study, and thus not addressed, included animated or video introduction to cancer treatment modalities and integration with electronic medical records and patient portal.

### 2.3. Focus Groups

The study team prepared a mock website for participants' review with new content and graphics (photographs of patients, computer users, and healthcare providers). A professional healthcare translation/interpretation company translated the mock website to Spanish. Focus groups were conducted to explore participants' previous experiences communicating about symptoms and quality of life issues, Internet use, and overall interest in electronic SQOL reporting and education. Participants were asked to evaluate the mock website for cultural sensitivity and appropriateness of terminology.

We planned to hold one initial focus group in Boston and two in Tampa. Instead, we conducted two initial focus groups in Boston and one in Tampa due to difficulty with recruitment in Tampa. Focus group meetings were conducted using an interview guide with specific open-ended questions that began with discussion of patient/caregiver experiences and moved to discussion of the mock website (Table 1). Bilingual, bicultural facilitators (authors Laura Gonzalez, Veronica Sanchez Varela, and Rosalyn Negrón) led the focus groups. Members of the study team observed and took notes; sessions were audio recorded and then transcribed and translated into English. The research team reviewed the transcripts, discussed all suggestions and recommendations, and prioritized revisions for implementation.

Focus groups were reconvened to evaluate the revisions and provide further feedback. A summary of the recommendations from the first round of review was presented to the reconvened groups and the revised ESRA-C was reviewed. Participants were asked to confirm that all of the recommended changes were implemented to their satisfaction and to provide additional suggestions.

### 2.4. Cognitive Interviews

Based on the suggestions of the key informant panel, a series of 36 cognitive interviews were conducted in Boston to assess understanding and ability to complete symptom and quality of life questionnaires included in ESRA-C. Patients, not caregivers, were invited to participate in cognitive interviews. Cognitive interviewing [47] was selected because it is a useful technique for evaluating health related educational materials and survey questions. Cognitive interviewing encourages participants to discuss how they arrive at responses, reflect on their interpretation of each question and response option, and discuss any problem they have in responding [47].

During audio-recorded individual interviews, patients were asked to evaluate SQOL questionnaires in Spanish using the “think aloud” method and interview probes. Existing Spanish translations of the questionnaires were used when they were available and others were translated into Spanish by professional translators for use in this study. The “think aloud” method was used because open-ended questions minimize the impact of interviewer imposed bias and interviewer probing allows for deeper insight into individual responses [48]. Participants were asked to discuss whether questions, vocabulary, and context were easily understood and make suggestions for revision. All interviews were conducted in a private room within the cancer center. The research assistant conducting the interviews also took written notes. Participants provided feedback on as many of the questionnaires as relevant to their diagnosis, disease status, and treatment history.

### 2.5. Cultural Sensitivity

*Cultural Sensitivity Assessment Tool*. Following the focus groups and cognitive interviews, final revisions were made to the mock website. The cultural sensitivity assessment tool (CSAT) [49] was then used as a formal measure of cultural sensitivity. The CSAT was originally developed to assess cancer education materials for African Americans. In this tool, cultural sensitivity has been defined as “an awareness and utilization of knowledge related to ethnicity, culture, gender, or sexual orientation in explaining and understanding situations and responses of individuals in their environment” [50]. When applied to evaluation of health related materials, it means that the message and content (1) is understandable to the intended audience, (2) reflects understanding of the cultural context of behavior, and (3) is congruent with cultural norms [51].

A document containing all Spanish ESRA-C assessment items and educational text along with the mock website URL was provided to three reviewers external to the study team who applied the CSAT. The reviewers were bilingual, bicultural professionals. Two reviewers, who were employed as patient navigators for Hispanic patients, had also participated in the key informant panel. The third reviewer was employed in a Hispanic community as a child and family welfare officer.

The CSAT is comprised of two evaluation sets, one for print and one for audiovisual (AV) material. We adapted the CSAT, drawing from both versions, to assess cultural sensitivity of a cancer education website with both text and audiovisual components for Hispanic users. Our final version included 56 items in three categories: format (7 AV items; e.g.,* the audiovisual medium is appropriate for the intended audience*); written/verbal message (11 print items, 11 AV items; e.g.,* the medical terms used in the verbal message are understandable to the intended audience*); and visual message (11 print items, 16 AV items; e.g.,* the visuals illustrate contemporary activities of the intended audience*), with each Likert-type item ranging from 4 (strongly agree that the information is culturally sensitive) to 1 (strongly disagree). Each category's scores were averaged across items and reviewers. Scores were tabulated and compared to acceptable benchmarks set by the CSAT authors [49] of 3.3 for audiovisual and 2.5 for written material categories.

### 2.6. Readability

*The Fry Readability Formula*. The Fry readability formula is a widely recognized formula for assessing readability of both English and Spanish materials and has been validated in both languages [52]. The Fry formula is especially useful in evaluating Spanish reading materials, because it takes into account the structure of the Spanish language, allowing for more polysyllabic words than English materials at the same reading level [45]. Following instructions for applying the formula, the study team selected three, 100-word passages for scoring from the three sections of educational text in the final Spanish mock website/content document:* Meet Your Care Team (Conozca a los miembros de su equipo médico)*, which introduces patients to the roles of various healthcare professionals in an oncology setting;* Getting Help (Cómo obtener ayuda)*, which coaches patients to engage in self-care for symptom and quality of life issues and to communicate problems to their providers; and* What can I do about fatigue? (*¿*Qué puedo hacer al respecto la fatiga (mucho cansancio)?)*, an activity-based intervention for cancer-related fatigue. Average numbers of sentences and syllables were derived for each section and plotted on the Fry graph to obtain reading levels.

### 2.7. Data Analysis

Descriptive statistics were used to evaluate demographic characteristics of participants. The anonymous focus group data were entered into a code-based data analysis software package (Atlas.ti). Members of the study team independently listened to focus group audio recordings, read observer notes, and reviewed English transcripts of each focus group. Bilingual research team members also reviewed the Spanish transcripts. Transcripts were analyzed to determine necessary revisions to ESRA-C that would improve cultural sensitivity and linguistic appropriateness. CSAT scores were reviewed and low scoring items identified. Fry readability levels were reviewed and areas scoring above a 6th grade level identified.

## 3. Results

### 3.1. Sample

Two initial focus groups were held in Boston, but recruitment in Tampa only filled one group. Following revisions to ESRA-C, two groups were reconvened in Boston and a new group was convened in Tampa. Individual interviews were also held with patients in Boston. A total of 51 individuals, 32 women and 19 men, participated in the study (Table 2). Approximately half of the participants in the study were from the Dominican Republic with the remaining reporting Puerto Rican, Cuban, Salvadoran, Guatemalan, Colombian, Ecuadorian, or Chilean heritage. Ages ranged from 32 to 86 with a mean age of 61 years.

### 3.2. Focus Group Results

*Initial Focus Group Meetings*. Participants described language related barriers to effective communication, including apprehension about saying things correctly in English, frustration when communication is difficult, and the need to rely on family members or professional interpreters to aid communication. Participants expressed the need to speak to their physician by telephone and shared frustrations about not being able to use the telephone to communicate because of language barriers. In spite of difficulties with communication, participants consistently offered praise for healthcare providers.

The patients described using the Internet to obtain general health information and to access personal medical records. They often relied on caregivers and family members to access the Internet and retrieve information. Typical access to the Internet was on a home computer rather than on a mobile device. Participants discussed use of the Internet for maintaining contact and socializing with others and some described how social media was used to update loved ones regarding their condition. Sharing information about cancer experiences with others via social media was described as a way to help others who may be going through similar experiences. The importance of being able to provide emotional support to others, as well as receive emotional support from family and friends, was a recurrent topic. Spirituality and faith in God were important coping strategies reported by participants. Participants reported that ESRA-C would be useful for helping patients know what to expect during cancer treatment. The following quotes are demonstrative of how participants perceived ESRA-C to be useful during cancer treatment.

*“I think this (ESRA-C) is a way to help people express what they feel and inform the doctor when they see him, to understand what's going on…because it is easier to express in writing all those concerns or feelings you have inside. This is a way to help communication.” *

*“It is wonderful what you are doing, it is so necessary because at any time of the day, you could sit in front of this computer and express what you're feeling, at that moment!”*

*“We are entering into a path, into a sort of dark tunnel without knowing anything but in this case…you can understand, and you are able to write it down.”*

Most participants stated that the list of symptom and quality of life issues was easy to understand. Suggestions for revision included adding specific skin-related symptoms such as dry skin and burns, adding more emotional descriptors (bad mood, tranquility), expanding definitions, and eliminating difficult-to-understand terminology. Some participants reported difficulty with reporting pain on a 0–10 scale and suggested the use of smiling or frowning faces as a gauge of pain levels. Some participants voiced misunderstanding one questionnaire's response option that read, “more than half the days” (of a week).

Perceived potential benefits of ESRA-C Spanish included improved patient-physician communication, ability to instantly report symptoms, and ability to anonymously report and receive information on subjects of a sensitive nature, such as depression and sexuality. Perceived potential barriers included concerns that the information from ESRA-C Spanish would not be communicated to doctors. Participants were concerned that some of the medical terminology, such as the words* fatigue* and* palliative care*, may be difficult for all patients to understand.

Participants in the initial three focus groups identified numerous areas for revisions to ESRA-C (Table 3). Participants expressed a strong preference for the content to be presented using first-person language, incorporating patient photographs, voices, and testimonials. The mock website was revised to include narration and presentation of content from a patient perspective. We also revised the text content to reflect suggested changes, revised the layout to further emphasize the main subject on each page with larger and bolder fonts, streamlined navigational elements, and added screenshots of the ESRA-C website to the narrated patient story to illustrate how to use the program.

*Reconvening the Focus Groups.* After suggestions from the initial focus groups were reviewed and revisions were made to the mock ESRA-C website, participants were invited back for a second meeting to evaluate the changes and make further recommendations. The participants in the second focus group in Tampa had not participated in the initial focus group because previous participants could not be contacted or were no longer available. All groups were provided with an overview of the revisions and presented with a mock-up of the revised version. Participants reported satisfaction with the revisions. They commented positively on the new patient-led narration, stating that the narrator was easy to relate to and encouraging and that the narrator gave important information about communicating symptoms to the clinical team. Further suggestions for revisions included adding information on nutrition, prevalence of symptoms, prevalence of cancer, and what to expect from chemotherapy and radiation therapy. Participants recommended that the program emphasize that every person's experience is unique.

### 3.3. Cognitive Interview Results

Analyses of individual interview responses (Table 4) indicate that participants had little difficulty with most items in most instruments. Participants identified specific issues with understanding terminology and had difficulty understanding response options for select items and questionnaires.

### 3.4. CSAT Results

Average scores for the two print materials categories of the CSAT ($\bar{x}$ = 3.2 across reviewers for written message, 2.9 for visual message) exceeded the minimum scores (2.5 of 4) recommended by the CSAT authors for acceptability [53]. Among the categories for audiovisual materials, the average scores in all three categories (3.0 for format, 3.1 for verbal message, 2.9 for visual message) fell below the recommended level of 3.3. Comments from the reviewers indicated that the lowest scores, related to visual message, were because one proxy patient presented in photos and voiceover could not represent the possible diversity of appearance (e.g., in skin color and face shape) of target users (Table 5). One reviewer stated “There is not one common physical feature for Latinos. We're a racially diverse group so that should be represented.” Most of the additional comments from reviewers (*n* = 15) suggested minor wording changes to questions in validated questionnaire translations or to educational text, such as the simpler word* debilidad* instead of* extenuación* in an item assessing fatigue.

### 3.5. Readability Results

The* Meet Your Care Team* content was scored at a 10th grade reading level. The* Getting Help* content was scored at a 4th grade, and the* Fatigue Activity Intervention* content was scored at a 6th grade reading level. We were able to achieve the targeted 6th grade reading level for cancer education materials in those sections of ESRA-C Spanish in which we engaged the expertise of a health communication writer. This writer revised the English text prior to translation and focused on enhancing readability through both lower reading level (as measured by readability formulas) and other factors such as chunking and bulleting text and formatting it with white space. The* Meet Your Care Team* section was developed last, and due to time constraints during the study we did not engage the involvement of the health communication writer. It had the highest reading level of all text scored.

### 3.6. Revisions to ESRA-C

Changes were made to ESRA-C as a result of key informant panel review, both sets of focus groups, and cognitive interviews.  Table 6 presents a summary of these changes and how each was evaluated. All suggestions made by participants or key informant panel member were considered by the research team and changes were made consistent with improving communication with healthcare providers, providing information on expected side effects of cancer treatment, and allowing patients to monitor changes in SQOL over time. Some suggestions from focus groups (e.g., newsletter, moderated chat) that were not in direct alignment with the goals of ESRA-C or which exceeded available resources were not implemented.

## 4. Discussion

In this study of the iterative content development of a web-based symptom and quality of life assessment and support intervention (ESRA-C) for Spanish-speaking patients with cancer, we found that patients, family caregivers, and professionals who work with them (key informant panel members) generally endorsed the concept and content of ESRA-C. When concerns about Internet access and acceptability were raised, most patients and caregivers indicated they would be able to use ESRA-C, with assistance from family members, if needed.

Other studies have also demonstrated similar preferences for Spanish language educational materials to be presented from a personal perspective with attention to family and spirituality as important cultural values. Consistent with our findings, others have described participants' privacy concerns with professional translators [54]. Researchers from Moffitt Cancer Center explored possible ways to increase Hispanic participation in clinical trials and also found preferences for the use of patient stories and experiences, depiction of family, and inclusion of elements representing spirituality such as a graphic depicting a family in prayer [55]. In a study adapting a colorectal cancer screening decision aid for Spanish-speaking Latinos, focus group participants stated a preference for personalism* (personalismo)* in having a patient character, rather than an anonymous narrator, be a guide to the medical information [56]. Others have described the importance of integrating these values into the care of Hispanics receiving palliative care [42], and our findings support the need to affirm and acknowledge these values across treatment settings and integrate them into patient education.

As in Ko and colleagues' work [56], we followed an iterative process to adapt an English-language web-based program in a way that preserves the benefits of the original intervention but adds cultural specificity, going beyond simple translation. We found that, in addition to patient/caregiver participation, input from a key informant panel of patient navigators and interpreters prior to the focus groups and formal evaluation of a mock website by expert reviewers identified aspects of cultural appropriateness and useful content complementary to that provided by patients/caregivers.

Application of the CSAT and Fry readability formula were considered necessary strategies for formally identifying issues of cultural appropriateness and usability. In a CSAT evaluation of a web-based prostate cancer prevention education program [40], average score for written messages (3.28, range 3.20–3.37) was comparable to the score for the Spanish ESRA-C (3.15), while average scores for visual message (1.36, range 0.96–1.76) were considerably lower than those for Spanish ESRA-C. A study of cancer-related educational materials distributed by the New Jersey Health Department [57] indicated that half of the materials used by the Department received scores at or below the acceptable value of 2.5 in one or more categories of the CSAT. Using the Fry readability formula, almost all of the materials (93%) were written at a level beyond the recommended 6th grade reading level.

### 4.1. Limitations

Due to convenience sampling of patients/caregivers from two institutions in Boston and Tampa and recruitment challenges in Tampa, most participants came from Boston and are not representatives of all Spanish-dominant cancer patients in the US. Over half of our participants were Dominicans, which is more reflective of the Hispanic population of Boston than the US as a whole [58]. Conversely, no persons of Mexican heritage participated in our study, although the majority of Hispanics in the US are of Mexican heritage [59].

Even though participants in this study reported being able to access the Internet, our sample may not be representative of other Spanish-speaking groups in the US. Internet use among Hispanics, particularly those who are Spanish-dominant, has been reported to be lower [12, 60] than what participants in our study described. Some of the participants did not access the Internet themselves, but instead younger family members accessed medical information from the Internet and passed it on to the older patients. This is consistent with studies showing that Hispanics of older age are less likely to use the Internet themselves [61].

Our measures of cultural appropriateness and reading level were limited. The CSAT was developed in the 1990s, one version for print and another for audiovisual materials. Neither instrument adequately measures interactive websites. It is widely acknowledged that aspects of medium and formatting affect readability, but tools assessing these factors are in development stages or not yet validated [62–64].

### 4.2. Future Research

In future studies, we will revise the website to include photos and voiceovers with a variety of patient characteristics, incorporate a moderated chat feature, and add a guided tour of how to use the website. We plan to include more resources for caregivers into ESRA-C. Research indicates that family preferences heavily influence treatment decisions, particularly in less acculturated Hispanics [65]. To address readability and appropriate wording, we will examine feedback from reviewers, engage a health communication writer in revising or developing new content, and reassess our choice of symptom instruments and external website links. We will incorporate resources specifically for caregivers and expand messaging on the diagnosis of cancer to address participants' concerns that newly diagnosed patients and others around them may view cancer as a death sentence.

## 5. Conclusions

The findings from this study provide beginning evidence that ESRA-C Spanish program will provide understandable and relevant information to Spanish-speaking patients with cancer and their caregivers. Assessment of cultural sensitivity supported acceptable levels of sensitivity and provided information about aspects of the intervention that could be refined to enhance cultural sensitivity. Our results provide important next steps for development of the ESRA-C Spanish program.

## Figures and Tables

**Table 1:** Focus group interview topics and prompts.

Round 1	
Communicating about symptoms and quality of life	(1) Tell us about a time when you told your doctor or nurse about a symptom that was bothering you—like trouble sleeping or being sick to your stomach. What was difficult or easy about this time?
	(2) How do you usually let your doctor or nurse know about these things? How does speaking Spanish affect what you do or do not tell your doctor or nurse?
	(3) Here is a list of symptoms and quality of life issues common for people during treatment for cancer. Is there anything missing? Is there anything on the list that is not easy to understand?

Using the Internet	(4) How often do you use the Internet, and how do you access it—for example, from a computer, your phone? Do you know how to access the Internet yourself, or does someone help you?
	(5) Are there websites or applications that you use to learn about health information or to track your activities, like exercise? What do you like about these websites or applications?

Responding to Spanish ESRA-C mock website	(6) Why do you think this website would, or would not, be interesting or useful for you?
	(7) Do you think most people can understand these questions clearly and give answers?
	(8) What do you think patients and their families would find most helpful about this information? How can we make it better?

Round 2	

Review of new patient narration	(1) How well does this new version meet the ideas you had about hearing from a patient's point of view?
	(2) What thoughts did you have about the patient shown in the pictures who was telling her story? How similar are her concerns to the ones that you thought about when you were going to start treatment?

Review of new website home page and introduction	(3) You saw the woman using the website to learn about getting help and then telling her nurse about problems she was having. How much does this encourage you to talk to your nurse or doctor?
	(4) Did we explain well enough for you to understand that the answers you give on this website will go to your doctors and nurses?

Review of messages in revised patient education and communication coaching	(5) If you put these three messages in order from most to least important for new patients to hear, what order would you put them in? Why?
	(6) Are there any words in the website that do not sound right to you, or do you think we could say better?

Working with interpreters	(7) If you have used professional interpreters, what have you learned about the process that you wish you had known before you started your treatment? If you have not, what would you like to know about using interpreters?

Emailing providers	(8) Have you had experience emailing your providers, and if so, how well did that work?
	(9) How would you feel about being able to email a question in Spanish to a translator or bilingual provider who could ask your doctor or nurse and reply to you?

Prioritizing future revisions	(10) The last thing we want to show you is a list of things that people suggested should be added to the website. If you like any of these ideas, or another idea you have, which of these should we work on first?

**Table 2:** Demographic characteristics and participant activity (*N* = 51).

	Boston	Tampa	Total
	(*n* = 45)	(*n* = 6)	*N* (%)
Type of participant			
Patient	36	4	40 (78.4%)
Caregiver	9	2	11 (21.6%)
Participant activity			
Focus group only	9	6	15 (29.4%)
Focus group and cognitive interview	20	0	20 (39.2%)
Cognitive interview only	16	0	16 (31.4%)
Gender			
Female	28	4	32 (62.7%)
Male	17	2	19 (37.3%)
Marital status			
Single, separated, divorced, and widowed	24	2	26 (51.0%)
Married/partnered	21	4	25 (49.0%)
Education			
<High school	22	0	22 (43.1%)
High school	15	0	15 (29.4%)
Some college	2	2	4 (7.8%)
College graduate	6	2	8 (15.7%)
School graduate	0	1	1 (2%)
Missing	0	1	1 (2%)
Nationality			
Dominican	29	0	29 (56.9%)
Puerto Rican	3	2	5 (9.8%)
Ecuadorian	4	0	4 (7.8%)
Colombian	3	0	3 (5.9%)
Salvadoran	3	0	3 (5.9%)
South American, not otherwise specified	0	3	3 (5.9%)
Cuban	1	1	2 (3.9%)
Chilean	1	0	1 (2.0%)
Guatemalan	1	0	1 (2.0%)
Race			
White	11	3	14 (27.5%)
Mestizo	13	0	13 (24.5%)
Did not select race option, wrote in Hispanic/Latino	14	2	16 (31.4%)
Missing	7	1	8 (15.7%)

**Table 3:** Qualitative analysis of focus group transcripts: topics, themes, and concepts.

Topic	Theme	Concepts
Internet use	Use of the Internet for cancer information	Survivorship Disease information Clinical trials Medication Nutrition Use of specific websites including Medline, WebMD, Facebook, and local patient portal
	For medical information	Test results Choosing a doctor
	Internet for social and practical purposes	Social networking and support News Manage finances
	Mode of Internet use	Do not rely solely on Internet for medical information Limited use of smartphones Primarily use computers at home, work, and at the clinic Need assistance from friends/family to use Internet

Communication with healthcare providers	Patient related barriers	Language barriers Difficulty discussing sensitive topics Nervousness hinders communication Prefer to use Spanish websites
	Provider related barriers	Providers unavailable for communication by phone/email

Appraisal of symptom and quality of life issues	Add/revise skin-related symptoms	Discoloration, skin irritation, dryness, and burns
	Add/revise emotional symptoms	Include bad mood, anxiety, motivation, peace, and tranquility
	Add additional symptoms	Runny nose, fever, problems with urination, weight gain, and headaches
	Symptoms for possible revision	Prefer “tiredness,” “weariness,” or “exhaustion” over fatigue Revise “poor emotional state” for clarity Specify type of “pain”

Feedback regarding Spanish ESRA-C	Perceived benefits	Improved communication with providers Can report symptoms and get information in private Helps to know what to expect “Real time” symptom appraisal Asks about suicidal thoughts and depression
	Perceived barriers to use	Medical terminology difficult to understand Wants to be sure healthcare team will get the information
	Suggestions for changes to assessments/responses	Prefer faces scale for pain assessment Question “never” as an appropriate response to SQOL Discussion of pain levels not clear (0–10 scale) Response of “more than half the days” difficult to understand
	Suggestions for additional features and messaging	More personal experiences from a patient perspective More familiar and identifiable icons Message that “cancer” does not necessarily mean death Search function Newsletter Dictionary of terms Email Moderated chat with other patients Caregiver assessments and resources Message that spirituality and faith are useful for coping with cancer Nutritional information Prevalence of symptoms Prevalence of different types of cancer What to expect from chemotherapy and radiation therapy

**Table 4:** Cognitive interview results: instruments, items, number participants per instrument, and issues.

Instrument	Items	Number of participants	Issues Identified
European Organization for Research and Treatment of Cancer-Quality of Life Questionnaire-C30 (EORTC-C30) [30] Spanish US Version 3	30	21	Difficulty responding to items pertaining to time or distance. Patients had concerns with shortness of breath item, relating it to asthma. Difficulty with responding to pain questions, associating the question with different kinds of pain. Difficulty with understanding the question of physical condition or medical treatment interfering with family life.

1-item neuropathy screener developed by research team	1	3	None reported or observed.

European Organization for Research and Treatment of Cancer Chemotherapy-Induced Peripheral Neuropathy 20 (CIPN20) [31]	20	19	Did not understand meaning of “adormecidos” (numbness) or “boligrafo” (pen).

Patient Reported Outcomes Measurement Information System (PROMIS) Pain Interference Short Form 6a [32, 33] Spanish v1.0	6	12	Did not understand meaning of “ocio” (leisure).

PROMIS Fatigue 7a Spanish v1.0 [34]	7	18	Did not understand meaning of “extenuación” (exhaustion).

PROMIS Depression 8a Spanish v1.0 [35]	8	12	None reported or observed.

Expanded Prostate Cancer Index Composite for Clinical Practice [36] (EPIC-CP)	16	6	Did not understand meaning of “almohadilla” (supposed to mean pad). The word “pad” does not have to be repeated in the response options since it was already in the question. Did not understand that men with prostate cancer could have these symptoms. Thought that “flujo” meant infection, not flow. Confused about the difference in response options (very small, small, moderate, or big problem). Confused about whether “funcion sexual” included both functional aspect and sexual desire.

EPIC-CP (revised translation)	16	9	Preferred the word “pañales” (diapers) rather than “protectores” (protectors). Did not understand meaning of “defecar” (bowel movement); preferred the word “evacuar.” Spanish slang terms for reaching orgasm (“venirse” or “acabar”), included in parentheses to assist low literacy readers, were offensive. Found the new word for flow (“chorrito”) appropriate.

Symptom Distress Scale [37] (SDS) Southwest Oncology Group Spanish version	13	14	Difficulty with similarity of the answer sets. Difficulty formulating response for insomnia (some with aches and pains keeping them up; they took a sleep aid to fall asleep). Not possible to be worried and not afraid.

Patient Health Questionnaire-Nine Symptom Checklist [38, 39] (PHQ-9) Spanish for USA	9	18	Difficulty understanding question and answers. Difficulty understanding either/or questions (e.g., trouble falling or staying asleep or sleeping too much); thought they should be asked individually.

Religion/Spirituality Questionnaire	4	12	None reported or observed.

Skin Problems Questionnaire	1	5	None reported or observed.

**Table 5:** Individual items with low scores (1 or 2) in the cultural sensitivity assessment tool (CSAT).

Category	Item	Reviewer scoring			Reviewer comments
		A	B	C	
Audiovisual: format	The sound effects make the subject matter more appealing.	n/a	3	**2**	Reviewer 3: when the provider enters an exam room in a sample visit, “knocking sound effect is not necessary.”

Audiovisual: visual message	The visuals (diagrams, pictures, and so on) reflect the variety of physical features (skin color, nose, lips, and so on) among Hispanics/Latinos.	**2**	3	**1**	

Print: visual message	The hair colors are representatives of the intended audience.	3	3	**2**	Reviewer 3: “There is not one common physical feature for Latinos. We're a racially diverse group so that should be represented.”
	The hair textures are representatives of the intended audience.	**2**	3	**1**	Reviewer 3: “Hair textures differ among Latinos.”
	The graphics accurately depict the physical features (breasts,…, etc.) of the intended audience.	**2**	3	n/a	

Scoring scale:

4 = strongly agree: the material *will absolutely be accepted* by the intended audience.

3 = agree: the material *will be accepted* by the intended audience but *could be better or could be improved* before it will absolutely be accepted by the intended audience.

2 = disagree: the material *will probably not be accepted* by the intended audience.

1 = strongly disagree: the material *will absolutely not be accepted* by the intended audience.

n/a = not applicable: the information *does not appear in the material* or *does not apply* to the material.

**Table 6:** Suggestions from key informants, focus group and cognitive interview participants, and study team members with resulting revisions and revision evaluation procedures.

Source	Suggestion	Revisions	Evaluation of revisions				
			Focus groups (RD 2)	Cog IWs	Reading level scoring	CSAT evaluators	Not yet (future revision)
Key informant panel	Assessment questionnaires should be rewritten because the reading level of some was too high.	Implemented cognitive interviews to assess patient understanding.		X			
	Educational text should be rewritten because the reading level was too high.	Revised all educational information on SQI self-care and communication coaching to a lower reading level.			X	X	
	Pop-up definitions should define terms that may be unfamiliar.	Added clickable pop-up in-context definitions.	X			X	
	Add detailed information on different treatment modalities.	Added general information on what to expect during treatment, including the importance of communicating symptoms to the care team.	X				

Focus groups	Need assistance with Internet.	Added screenshots illustrating use of ESRA-C.	X			X	
	Concerns about confidentiality with interpreters; unclear on role of some care team members.	Revised and expanded description of clinical team to include interpreters and give more information on each role. Explained that translators are professionals who have an obligation to keep private health information confidential.			X	X	
	More information on specifics of what to expect during treatment wanted.	Added links to information about treatment (surgery, chemotherapy, and/or radiation therapy) on the Dana-Farber website which can be customizable to institution.				X	

Focus groups	More information on nutrition wanted.	Link users to reputable web resources of appropriate reading level on nutrition for people undergoing cancer treatment.					X
	Add faces to pain numeric rating scale.	Adapt version of faces scale to be consistent with the style of the existing interface.					X

Cognitive interviews	Specific terminology or question structures were difficult to understand.	Revised translation of one questionnaire and noted other questionnaires for future revision or substitution.					X

Research team	Simplify layout and interface.	Revised layout and interface: larger, bolder fonts, fewer navigational links.	X			X	
	Add module on managing fatigue.	Added information on exercise, including instructions and an activity diary, for fatigue management.			X	X	

Reading level scoring	One section was scored at a 10th grade reading level and should be simplified.	Revise this section and examine reading level of linked web resources.					X

CSAT reviewers	About 15 specific suggestions for wording changes or additional content were made.	Review and consider implementation of these changes.					X
	Proxy patient shown was not representative of Hispanic patients.	Consider adding photographs of more patients and families.					X

Note: CSAT = cultural sensitivity assessment tool [40].
